# The plight of Ukrainian refugees staying in Sweden under EU:s mass refugee directive: a brief trauma-focused, participatory, online intervention as a pilot feasibility study

**DOI:** 10.3389/fdgth.2024.1461702

**Published:** 2025-01-24

**Authors:** Solvig Ekblad, Oksana Gramatik, Yuliia Suprun

**Affiliations:** Department of Learning, Informatics, Management and Ethics, Research Group Cultural Medicine, Karolinska Institutet (KI), Stockholm, Sweden

**Keywords:** Ukrainian, trauma, refugees, mixed-methods, education communication media, co-creation

## Abstract

**Background:**

Ukrainians staying in Sweden under the EU mass refugee directive may face challenges due to traumas caused by the invasion that started on February 24, 2022. Part of an European Social Fund (ESF) project, our study showed that a brief trauma-focused group intervention onsite helped to increase health and mental-health literacy. The intervention has not yet been adapted online.

**Methods:**

This pilot study during six months aimed to investigate the feasibility, acceptability and outcome in perceived trust, anxiety/stress, and perceived health after this brief trauma-focused group intervention online. A second aim was to observe perceived acceptability of the group intervention with different ways of online intervention. Local coaches, interpreters, the authors, and local experts participated. A mixed-methods design with participatory methodology and evaluation were used. Data was collected with a short questionnaire in Ukrainian. Additionally, at the end of each set, we orally asked about perceived trust and integrity. There were six sets of five group sessions per set, a total of 30 sessions. Each group met online five times for 2 h, a total of 10 h excluding pre- and post-assessment. Breathing exercises sought to reduce stress among the participants.

**Results:**

The group intervention had both strengths and limitations. Baseline data were obtained from 136 participants, mostly females (75.7%). Answers to pre- and post-questionnaires showed that perceived anxiety/stress was significantly reduced (*N* = 91, chi-2 20.648, df = 6, *p* < .02). Perceived health significantly improved between pre- (mean 63.6) and post (77.2) (*N* = 77, *t* = −8.08, df = 66, *p* < .001). Older participants were vulnerable with higher stress and lower mean perceived health after the intervention. Four out of ten needed individual psychosocial support online.The participants' open questions were analyzed with qualitative content analysis, giving five general categories and 25 sub-categories and the theme “Strong efforts to cope with Swedish system”.

**Conclusion:**

Trust and reduced anxiety level changed after the intervention and a combination online of small, closed group meetings with the possibility of personal acquaintance, trust and individual follow-up psychosocial support for those in need to be paid attention to for future refugee support services, particular an online format.

## Introduction

Since February 24th, 2022, many Ukrainians, mostly women and children, have sought refuge in other European countries, in the worst migration crisis in Europe since WW II (1). Sweden has received around 40 000 Ukrainians. The migrators were forced to move to maintain personal safety, i.e., not for better economic, educational or work opportunities. At present, their stay is restricted to March 4, 2026, and many are concerned that the war will still be ongoing, leaving them insecure about returning. Stress and worries are common among Ukrainian refugee crisis as they lack factors that may create a sense of stability and calm, such as a sense of belonging. According to Kaufman, Bhui and Katona (2), “this crisis requires international support and a global response, as hosting countries have specific competencies and capacities” (page 1).

Refugees' mental health needs are greater during resettlement and yet these may go unnoticed or untreated due to challenges faced by both the refugees themselves and by their service providers (3). These authors mention stigma and shame, as well as cultural and language barriers and other psychosocial challenges (e.g., job, studies, transport, poverty, lack of orientation and information). Further, refugees reportedly lack trust in the local authorities, while these may have previous negative experience of authorities in the refugees' home country (3). In addition, when health providers can speak the refugees' native language, the latter are more likely to trust health providers and care when needed (4).

Healthy lifestyle behaviors are often overlooked during war but crucially important with the risk of intergenerational effect and communication challenges between individual, family, and society. A cohort study shows that post-traumatic stress syndrome (PTSD) in refugees is associated with harsh parenting styles, leading to adverse effects on their children's mental health (5). Programs for parents to increase their health and mental health literacy will be important primary prevention tools to reduce the transgenerational effects.

To our knowledge, the article by Buchcik, Kovach and Adedeji (6) is one of the earliest studies of the mental health of Ukrainian war refugees since the invasion. The study was based on a sample of 304 individuals surveyed in Germany. It showed that severe psychological distress, severe depressive symptoms, and anxiety prevail among a significant portion of refugees. The results further identify the vulnerability of women refugees to poorer mental health outcomes. In line with this observation, another study also reported that Ukrainian refugees staying in Poland showed severe depressive and anxiety symptoms at a comparable level (7).

About one-third of refugees develop PTSD after war trauma, but many have multiple traumas, which is why the number can be higher (8). People who experience trauma are more likely to die younger of all causes, develop chronic illnesses and severe social disabilities (9).

Cumulatively, these facts demand a new emphasis on increasing primary health promotion prevention. In our research, perceived stress/anxiety decreased and perceived health and trust increased among Ukrainian refugees direct after the five weeks trauma-focused group intervention with participatory methodology. We also ran a short breathing relaxation exercise which helped the individual recover psychologically (10).

The geographical spread of refugees and other migrants limits their access to culturally and linguistically appropriate services for those in need. A relevant implication would be digital (mHealth) interventions as new tools for addressing a spread population of refugees and other immigrants and their needs for facts and information to improve their mental health and well-being.

Digital interventions (mobile applications, tele-counselling, online therapy and e.g., mobile phones) allow people to access interventions without the need to attend a clinic or health-care facility (11). A systematic review of digital interventions for the mental health and well-being of international migrants concluded that few digital interventions have been designed to address the mental health and well-being of international migrants (12). Their importance lies in increasing access to mental health services, preventing daily-wage loss, reducing language and literacy barriers and being available to more people (12). Further, digital interventions are accessible to people with special needs and the opportunity to receive assistance in remote parts of the country and during constant movement.

To our knowledge no other brief trauma-focused group online interventions have yet been performed for members of the Ukrainian refugee community. Using interactive training for cross-cultural trauma-informed care in the refugee community, Im and Swan (3) showed that face-to-face interaction and mutual learning facilitated building relationships and trust among participants, and especially those between refugee/immigrant-community leaders and service providers. Given the short project time (six months) and the distances between “our” municipalities, we wanted to explore whether trauma-focused group intervention (10) on Teams would be feasible and acceptable among Ukrainians in Sweden under the EU mass refugee directive. Thus, the findings could help improve trust and primary prevention among possible dropouts.

### Aim

This pilot study aimed to investigate the feasibility, acceptability and outcome in perceived trust, anxiety/stress and perceived health after this brief trauma-focused group intervention online. A second aim was to observe perceived acceptability of the group intervention with different ways of online intervention.

## Material and methods

### Context

The study was part of a second social-fund project (Fast Care Ukrainakompetens 23-043-568, www.invandrarindex.se) and was performed in the same five Västernorrland communities as the first project: Härnösand, Sollefteå, Sundsvall, Ånge, and Örnsköldsvik (10).

The STROBE Statement checklist (*Strengthening the Reporting of Observational Studies in Epidemiology* (13) was used as a guide to reporting.

### Procedure

We used the ADAPT theoretical model developed by Silove (14) and a participatory methodological approach (15) according to the five themes from the first study (10) (Box 1). In view of the actual needs of the potential participants and their longer stay, the content of respective themes were a little revised. The team now included the Migration Agency, as participants had many questions regarding EU rules governing the time limitation of their stay. Another area of interest concerned the participants' work options. We invited a representative from the Swedish Public Employment Service and one from the municipality. The first author continued to supervise the second and third authors performing and following up the group intervention and giving individual psychosocial support online for those subsequently needing it (10). We had weekly regular, or more frequent, online contact with local Ukrainian coaches. The first author met the five local coaches nearly every third week online for reflection.

BOX 1 **Themes of intervention sessions online led by different local team experts (Could be in different order locally and according to participants' length of stay).**

**Theme 1. Stress, recovery and repatriation stress** Aim: Raise awareness among refugees about the stress associated with migration and repatriation and provide them with practical tools to manage stress and promote psychological recovery (e.g., resilience techniques, cognitive behavioural therapy technique, cbt). Develop stress management skills among refugees: teach them relaxation techniques, breathing exercises, physical activity and healthy eating, and help them find social support to cope with the difficulties associated with migration and the experience of war. Experts: The three authors online. Main content: (1) Identify and understand the different types of stress that refugees may face, including stress associated with fleeing war, adapting to a new culture, and potential stress upon returning to their home country. (2) Discuss the physical and emotional symptoms of stress and their impact on daily life. (3) Introduce effective coping strategies for stress, such as relaxation techniques, breathing, exercise, and healthy eating. (4) Prepare refugees for potential challenges during repatriation and provide them with tools to make decisions about their future. (5) Answers from experts to questions from participants. Interpreter: the second and third authors online and a local interpreter online. **Theme 2: The Swedish Public Employment Service** Aim: To provide refugees from Ukraine with the necessary knowledge about the Swedish labour market so that they can successfully integrate into it and find work. Expert/s: Local experts in Swedish labour market (Observation of the three authors online). Main content: (1) Introduce the employment process: Explain how to look for a job in Sweden (online platforms, agencies, direct contacts with employers). Describe the process of applying for a job and going through an interview. Explain what a CV and cover letter are and how to write them correctly. (2) Help understand the education system and recognition of diplomas. (3) Introduce the rights and responsibilities of employees: Explain the main provisions of Swedish labour law. Describe the different types of employment contracts and their features. Explain what trade unions are, what functions they have, and how to join a trade union. (4) Help adapt to the work culture in Sweden. (5) Answers from local experts to questions from the participants. Interpreter: local interpreters onsite and online. **Theme 3: Human rights** Aim: To provide Ukrainian refugees with comprehensive information about their rights, obligations and opportunities in Sweden so that they can effectively integrate into the new society and plan for their future and the future of their children. Experts: Local assistants from the the Migration Agency online (Observation of the three authors online). Main content: (1) Rights and obligations of refugees in Sweden. Temporary Protection Directive: A detailed explanation of this directive, what rights it grants and what restrictions it imposes. Social guarantees: Information on social benefits, health insurance, education for adults and children. Work rights. (2) Living in Sweden. Rules for obtaining housing and changing housing. (3) Rights of mothers and children. Support for pregnant women. Education of children. Social services for families. (4) The future in Sweden after the war. Options for further residence. (5) Answers from experts to questions from participants. Interpreter: Local interpreter online. **Theme: Swedish healthcare** Aim: Provide Ukrainian refugees with the necessary information about the healthcare system in Sweden so that they can receive quality medical care in a timely manner and understand their rights. Expert/s: Swedish and Ukrainian medical doctor online (Observation of the three authors online). Main content: (1) Introduction to the Swedish healthcare system: a brief overview, basic principles. (2) Primary healthcare: family doctors, medical centers, making appointments. (3) Secondary health care: hospitals, specialists, hospitalization. (4) Emergency health care: calling an ambulance, providing first aid. (5) Patients' rights in Sweden: basic rights, how to protect them. (6) Health care for refugees: features, available services. (7) Medical personnel: who is who, how to interact. (8) Answers from experts to questions from the participants. Interpreter: Local interpreter online. **Theme 5: Swedish enterprise—the municipality** Aim: To help refugees successfully integrate into the Swedish labour market by providing them with practical knowledge about the employment procedure in Swedish municipalities. Experts/s: Local experts from labour market online (Observation of the three authors online). Main content: (1) To introduce the recruitment procedure in municipalities: explain how to search for vacancies, how to apply, what stages the selection process goes through. (2) To provide recommendations on writing a resume and a motivation letter: teach how to adapt your documents to the requirements of the Swedish labor market. (3) To give advice on successfully passing an interview. (4) Answers. From expert/s to questions from participants. Interpreter: Local interpreter online.

### The intervention

During the monthly information meeting in the project in each municipality, potential participants received by the local coaches written and oral information in Swedish, Ukrainian or Russian about the group intervention, in which participation was voluntary. Those who were interested signed consent forms including contact information and gave it to the local coach. The local coach sent the contact information to the second and third authors who informed them again about the intervention and concrete time and place.

The interpreter, online or using a mobile phone, spoke Ukrainian, Russian and Swedish. Where relevant, the local coach asked participants whether Russian was accepted before the session. Each group included about 12 participants, but given online and geographical constraints, outside our control, there could be 20–30. In addition, a small and closed group of 12 deaf participants worked on Zoom with an interpreter in Kyiv (these participants knew Russian sign language but not the Ukrainian equivalent).

As in earlier projects with such trauma-focused group intervention, our groups were closed to increase trust during each session. We followed eight basic principles: (1) The interpreter had a duty of confidentiality and translated everything said in the room. (2) Everything said in the room stayed in the room. (3) Mobile phones were switched off. (4) Concentration on the here-and-now and feeling mentally good. (5) Religion and politics as well as economy and housing were discussed only in other contexts. (6) Hands were raised for questions and comments (7) “It's a closed group-no one comes or goes”. (8) No information to authorities (privacy was the main policy, but exceptions were e.g., reporting obligations under Swedish legislation governing professionals).

The two-hour sessions included a short break for refreshments. The first session included facts from the team expert and during the second session, the participants were invited to ask questions. At the beginning and end of each session, there was a short breathing relaxation exercise supervised online by the second and third authors to reduce participants' stress. Research and our first study indicated that this deep breathing technique can promote an improvement in self-reported evaluations of mood and stress and of objective parameters (10).

### Study design and data collection

This mixed-methods study resembled earlier projects with this intervention [(10), page 4]. So far, no reliability and validity measures have been performed because the projects were limited in time and were explorative for different language groups.

The *feasibility and acceptability* outcomes were based on recruitment of all potential participants and drop-outs.

The following self-rated measure outcome points were defined:

• Baseline measurement of severity of anxiety/stress symptoms related to perceived health: The anxiety/stress question had three response options (none, to a certain extent, and to a great extent), with subsequent open-response questions. Initially: “if you feel anxiety/stress, what do you feel is the cause of this?” … “If you feel anxiety/stress, what do you usually do to feel better?” Further, estimated perceived health was assessed on a visual analogue scale where 0 represented the worst possible perceived health and 100 the best.
• Pre-post perceived health and mental literacy: “If you feel anxiety/stress, what do you experience after the group intervention are the causes of your anxiety/stress?” …“What knowledge and tools have you gained during the group meetings to feel less anxiety/stress and feel better?”
• Just at the end of the intervention, the follow-up symptom-severity measure of perceived anxiety/stress: We used the same three-option question regarding anxiety/stress as for the baseline measurement to assess symptom reduction. Estimated perceived health was assessed again on a visual analogue scale where 0 represented the worst possible perceived health and 100 the best.
• Group dynamics: Group dynamics were operationalized by estimating activity by the participants who asked questions and made comments in each meeting.
• Follow-up with individual psychosocial support online under supervision.

### Online interaction

We used three ways of online interaction:

1. Group meetings in small, closed groups with the possibility of personal acquaintance (Teams)
2. Large group online from different municipalities (Teams)
3. Individual follow-up, psychosocial support for those in need (Mobile)

### Participants

#### Inclusion criteria

The minimum age for participation in the study was 18 years old. The participants were Ukrainian people residing for a fixed period in the five Swedish municipalities under the EU mass refugee directive. During their stay they were not allowed to seek asylum, but currently had permission to stay until 4 March 2026.

One hundred and thirty-six Ukrainians, mostly women, participated between 1 October 2023 and 31 March 2024.

#### Exclusion criteria

Excluded were newcomers from other countries and Ukrainians who had not arrived under the mass refugee directive and lived in other Swedish municipalities.

### Translation and back-translation as a culturally adapted guideline

The written information, consensus and pre- and postintervention questionnaires were translated from Swedish into Ukrainian or Russian and back. They were also culturally adapted and discussed by the three authors according to WHO guidelines (16).

### Data analysis and interpretation

*Quantitative data* was analyzed using the Statistical Package for Social Sciences (SPSS) program version 28.01.1. Demographic and other background independent variables were age, number of children, number of months after arrival, number of moves in Sweden for Migration Agency reasons, country of birth, mother tongue, status, work in Ukraine and work in Sweden. Baseline characteristics were analyzed using descriptive statistics (frequencies, means, min-max, and standard deviations).

*Dependent variables*: The anxiety/stress question had three response options (none, to a certain extent, and to a great extent). Perceived health was assessed on a visual analogue scale where 0 represented the worst possible perceived health and 100 the best.

Pre-post comparison used statistical differences (*p* < 0.05) and correlation with background data (We used Spearman correlations because the data distributions tended to be slightly skewed); the chi-2 test when comparing two categorical variables and paired-samples and the *t* test for determining whether there was a significant difference between means T1 and T2.

Qualitative content analysis as outlined by Graneheim and Lundman (17) was used for the answers to the open questions. The entire data transcribed by the second and third authors was read and re-read repeatedly by the three authors. Condensed meaning units were sorted into codes containing the core messages. The codes were revised and sorted into preliminary themes and sub-themes. To optimize validity, every author independently read the transcribed material and the three authors discussed the material until agreement was reached.

### Ethical considerations

The first project (10) was approved before start by the Swedish Ethical Review Authority (2023-00092-02). The six-month time limitation prevented us from applying for new ethical approval; however, we used the same design, and all participants gave their written informed consent after introducing them to the study and in accordance with the Declaration of Helsinki. Participants were provided with information regarding their anonymity and secure data processing. Participants were also informed about their right to withdraw their participation at any time without explanation. In the presentation of the results, all quotes are anonymized.

Further, as we were investigating performance online, we ran a pilot study on Ukrainian participants arriving in Sweden under the mass refugee directive with fixed permission to stay. They were not patients. In an analysis of a review on research ethics and refugee health identified several complex ethical challenges in conducting refugee health-related investigations and found yet few noted whether refugee representatives had an opportunity to review investigational protocol. Cultural considerations were in general limited to gender and religious norms (18). In our study we had the ambition to overcome these challenges as follows.

By increasing health and mental health literacy we sought to detect those with high anxiety/stress and perceived illness and to offer knowledge and tools to increase trust and perceived health and mental health literacy. This might, we hoped, prevent participants from drop-outs. Where any participant needed professional psychosocial support, we had the competence and were prepared to advise them to contact the local primary health care. Another possibility was individual follow-up online (mobile) with the second and third authors, supervised by the first author.

## Results

### Participants characteristics

The group intervention included six sets of five group sessions per set (see Box 1)*,* a total of 30 sessions. Individual mental support for deaf people took place via WhatsApp and Telegram through typing. During the six-month study period, 136 attended the start of the six sessions. Fifty-four were followed up online for psychosocial support after the intervention, i.e., 39.7% of the total participants.

The results (T1 and T2) of the dropout analyses (*N* = 58) showed significant differences between those who answered the questions: more married people (*p* < .001), more Migration Agency movements in Sweden (*p* < .001), some baseline stress/anxiety (*p* < .001) and perceived higher perceived health after the group intervention (*p* < .007).

At baseline, most of the participants were women (103, 75.7%). Nearly half the participants were married (65, 47.8%), about one-fifth (23, 23.2%) were unmarried. Just over one-tenth were divorced (15, 11.0%) and just under one-tenth (9, 6.6%) were widows. The average age was 44,7 years (range: 18–75; S.D.12.28). The participants had between no and eight children (range: 0–8; mean 1.59, S.D. 1.20). They had spent on average 19 months in Sweden (range 1–48 months) and had in general moved “nearly twice” (1.7 times) (S.D. 1.55) due to Migration Agency decisions; but the frequency ranged between zero and nine times.

The most common mother tongue was Ukrainian (95, 69.8%), followed by bilingual Ukrainian and Russian (20, 14.7%), and Russian (18, 13.2%). Just under nine of 10 were born in Ukraine (121, 90.0%) and the others in Russia or the former USSR.

Before arrival, all participants had been working in Ukraine, in for example industry, health care, and different kinds of service. In Sweden, after arrival little more than two-thirds had no paid job (95, 69.9%). About 30 per cent (41, 30.9%) of the employed worked unqualified in e.g., hotels, car wash or restaurants.

### Measurement of anxiety/stress and perceived health (T1)

At baseline, T1 (*N* = 136) just over one-fifth (30, 22.1%) felt no anxiety/stress; six of ten (81, 59.6%) answered that they felt anxiety/stress to a certain extent and under one-tenth (12, 8.8%) largely felt anxiety/tress before the start.

At T1 (*N* = 121) the participants noted their present health on a visual analogue scale from 0–100, answering on average 62.7 (range 10–100, S.D. 21.28).

Before the intervention, participants reported the causes of anxiety/stress as

“War, moving, changes in the external environment, uncertainty about the future.”
“Somatic, psychological and social.”
“The war in Ukraine, university exams: I don't know what will happen to my future.”
“Worried about those people who remained in Ukraine, especially those who are currently fighting.”
“Responsible for my granddaughter, with whom I came to Sweden.”
“Bad finances, fewer friends, difficult adaptation, no confidence in the future, speech barrier.”
“Want to stay in Sweden, and get an education, but worried that it won't be easy because the Government is preparing a program for us to return home. I have nowhere to return to. Now I have a new life here.”
“Don't know the language, but it's hard for me to learn. I feel stressed …every day I have had bad news from Ukraine.”
“Don't have work, unemployed. Misunderstanding in the family.”

The participants with perceived stress/worries before the intervention replied that they did the following to feel better:

“Communication with friends and loved ones. I kiss my daughter and hold her.”
“Crochet and knit and drink coffee.”
“Communication with friends, meetings at the group intervention.”
“Follow a routine, physical activity, psychosocial practice.”
“I talk to my mother, watch TV series, make love.”
“Try to think positively, try to change attitude towards the situation.”‘
“Take antidepressants and drink a lot of water.”
“Eat chocolate, candy, drink soda.”
“Control my thoughts, listen to music, light candles, call a friend, pray.”

### Symptoms of anxiety/stress and perceived health (T1-T2)

Directly after T2, nearly half (42, 46.2%) felt no anxiety/stress, five out of ten answered that they felt anxiety/stress to certain extent and a few (3, 3.3%) that they largely felt anxiety/stress directly after. Among those who rated both at T1 and T2 (*N* = 91), perceived anxiety/stress was significantly reduced (chi-2 20.648, df = 6, *p* < 002). At T2 (*N* = 91) perceived health was on average 75.4 (range 10–100, S.D. 17.7). Perceived health significantly improved between T1 (mean 63.6) and T2 (mean 77.2) (*N* = 77, *t* = −8.08, df = 66, *p* < 0.001).

After the intervention, the participants perceived anxiety/stress as caused by:

“Somatic, mental and social problems.”
“Uncertainty, I can't find a job, I can't learn the language.”
“I am afraid for my life; I want to return to Charkiv. Feeling stressed by bad conditions in Sweden.”
“I worry about my relatives in Ukraine.”
“Worry about children in Ukraine.”
“Uncertainty, nowhere to return to. I can't find a job.”

The participants answered that during the intervention they had received the following knowledge and tools to reduce perceived stress/worries:

“(Learned to) control stress and anxiety, feel self-confidence, find strength and distract myself for a walk. Thank you for individual support.”
“Use psychological practice to deal with stress.”
“Was able to get answers to my questions to the Migration Agency and the Swedish Public Employment Service.”
“Breathing techniques, physical exercise, returning to the here-and-now.”
“Have learned a lot of new information for myself. I can better apply methods for calming myself after anxiety. Very interesting.”
“Breathing practice. Have got a lot of information about employment and social life.”

### Spearman's correlation coefficient

There was a significant negative Spearman correlation between age and perceived health before the intervention (−0.257, *p* < .001), i.e., the older in age the smaller the mean of perceived health, with significant positive correlation with perceived stress/anxiety after (0.450, *p* < .0.01) and negative between perceived health after the intervention (−0.331, *p* < .01), i.e., the older in age the higher the stress and lower the mean perceived health after the intervention. There was a significant negative correlation between perceived health before the intervention and perceived anxiety/stress after the intervention (−0.451, *p* < 0.01) and a positive correlation between perceived health before and after the intervention (0.697, *p* < 0.01).

### Group dynamics during intervention online

The three authors observed that the group dynamics during the online sessions, when all municipalities were together, reduced possibilities for individual questions. Individual support online after the intervention with the small group seemed to be acceptable and safe and gave trust as the second and third author talked individually, being recognized from the online group intervention. Further, the local coaches sometimes had technical problems during the session, and this may have increased stress among all involved and reduced feasibility. Interpretation was online for geographical reasons, but this could have reduced clear communication with *all* the participants. Other obstacles may have been feasibility, acceptability and outcome, and these need further investigation:

- The participants in most cases did not know each other (five communities at once).
- Safety and trust: the rules/policy of the group and the confidentiality of all information were explained before each meeting, but confirmation was difficult to get.
- Primary tension was reduced through initial and concluding breathing practice led by the second and third authors. However, while participants found this very useful in between the meetings, not everyone felt safe doing it in front of people they did not know.
- Emotional expression: Some participants were hesitant to express their feelings and thoughts, and this led to a less deep understanding of emotions and contribution to personal growth.
- The participants had difficulty feeling free to reflect during the meeting. They were invited to write down their questions to “their” local expert. However, it seemed that they were unable to ask questions orally during the group interaction, and we received very few questions for distribution to the local team expert, who would reply at the next group meeting.
- Support and compassion: The participants indirectly offered each other support and compassion. The nature of the group did not allow people to feel understood and cared for but created a limited sense of belonging and reduced feelings of isolation.
- Confidentiality and non-judgment: Confidentiality was quietly enforced within the whole group, though an atmosphere of non-judgment was very difficult to cultivate. This made it more difficult to allow participants to share their vulnerabilities without fear of criticism or judgment.

### Thematic analysis of participants' questions

Table 1 presents qualitative results from qualitative content analysis of participants' questions in dialogue with the respective local team expert during each session. They were written down by the second and third authors. One theme was highlighted, namely “Strong efforts to cope with Swedish system”. The five categories were: Strategies for reducing stress and illness; Ignorance of the Swedish labour market; Similarities & differences in the processes of mass refugee directive and asylum protection; The meaning of needs-based care; and High motivation to enter the Swedish labour market. For each category there were five subcategories of the questions raised by the participants.

**Table 1:** Qualitative content analysis in five categories (columns) and five sub-categories of the questions raised by the participants under the EU mass refugee directive (*N* = 136; number of intervention sessions 30). New questions added during the dialogue with each local expert.

1. Strategies for reducing stress and illness	2. Ignorance of the Swedish labor market	3. Similarities & differences in the process of mass-refugee directive and asylum protection	4. The meaning of needs-based care	5. High motivation to enter the Swedish labour market
1.1 Evidence-based knowledge on freeze, panic attack, loss of control (aggression) and sleep problem	2.1 Registration at age 65, sick leave and when child is sick, disability confirmation procedure. Daily allowance when leaving Sweden	3.1 Each individual is responsible for their own financial obligations. Payment conditions for housing when job in another city. School payment	4.1 Difference between insurance medicine and regular medicine. Rationale for 5 Covid vaccinations. Reimbursement for medical expenses. Emergency need at night	5.1 To write a motivation letter and CV
1.2 Prevention of social media addiction	2.2 Translation of diploma, equivalence	3.2 Open a bank account	4.2 Dental problems and payments	5.2 Interpretation of “no feed-back” from employers
1.3 Supporting a withdrawn child	2.3 Register at Unemployment office on gaining employment	3.3 Assistance of deaf and mute children	4.3 Options to undergo preventive medical examination. Gynaecology test. Care assistance after operation, rehabilitation hotel	5.3 Rules for permission to stay after end of war
1.4 Compensating a child for missing a father or close friend	2.4 Age of child to enter labour market, minimum salary	3.4 Ukrainian driving license in Sweden	4.4. Steps to be taken when child has worms or lice	5.4 Requirements for getting permanent work rather than <6- month contract
1.5 Quit smoking	2.5 Difference between permanent contract & seasonal	3.5 Options to obtain Swedish citizenship	4.5 Treatment of autistic children	5.5 Possibility to establish private company

One theme was highlighted, namely “Strong efforts to cope with Swedish system”. There were five categories: Strategies for reducing stress and illness; Ignorance of the Swedish labor market; Similarities & differences in the processes of the mass refugee directive and asylum protection; The meaning of needs-based care; and High motivation to enter the Swedish labour market.

## Discussion

This pilot study aimed to investigate the feasibility, acceptability and outcome in perceived trust, anxiety/stress and perceived health after this brief trauma-focused group intervention online. A second aim was to observe perceived acceptability of the group intervention with different ways of online intervention. The intervention had both strengths and limitations. At the conclusion of the intervention online, we asked about trust and integrity: the intervention online was feasible, but perceived trust and integrity remained low. The Spearman's correlation coefficient showed that the older in age the higher the stress and lower the mean perceived health after the intervention. A study in older Ukrainian refugees of war in Poland showed that the estimated data indicated that the disease burden of older Ukrainian refugees is considerable which pay attention to financial and logistical impacts on countries receiving the target group (19).

Qualitative content analysis of the participants' open questions in dialogue with the respective local team experts showed that the content of the questions had changed in comparison with the first study (10). The present study prompted many questions to the Migration Agency and to the Swedish Public Employment Service. Further, the medical questions to a doctor concerned disabilities, treatment and costs. This was also interpreted similar in the theme of the qualitative analyses which was “Strong efforts to cope with Swedish system”. The stress and recovery category included questions regarding strategies for reducing stress and illness. For this reason, the content of such a group intervention needs to be personalized and flexible in view of the specific needs.

The combination of small-group meetings and the possibility of personal acquaintance and individual follow-up and psychosocial support for those in need showed perceived acceptability. Our results support Bergevi et al. (20) systematic review regarding user perceptions of eHealth and mHealth services promoting physical activity and health diets shows that these services provide value but need to be tailored to individual needs. However, not intended to be used as a substitute for professional services, a scoping literature review and app evaluation shows that mHealth apps have the potential to foster a significant reduction in symptom severity for PTSD, anxiety, depression and other mental health illness (21). Our findings give support to improving adherence to personalized, individual psychosocial help after trauma-focused group intervention in small groups, with the possibility of personal acquaintance.

International research shows that about one-third of refugees develop PTSD after war trauma, but many have multiple traumas, so the number can be higher (8). The needs exceed the available resources. Swedish healthcare lacks resources and competence to accommodate a third of the Ukrainians as well as other group of newcomers (asylum seekers and refugees) with estimated need for health and mental health literacy. In the present study, four out of ten (39.7%) had access to individual psychosocial support online (mobile) from the second and third authors, and their perceived trust was satisfactory. Therefore, a trauma-focused short and closed group intervention such as the present constitutes primary prevention in combination with individual psychosocial support online.

### Strengths and limitations

The first strength of the study is the need-based trauma approach, plus the experience of the first project with similar target groups (10) by revealing a need for a scientific and cultural, methodological participatory approach. and refugees in Sweden across diverse domains (Box 1) including psychological and social aspects. The confidence of the participants was maintained with participatory methods (15). Participation was voluntary; consent was conditioned on anonymity and the presentation of results on group level.

The second strength is that the second and third authors have the same background and languages (Ukrainian and Russian), were educated in psychology before arrival in Sweden and were regularly supervised by the first author. As in the first study, they telegrammed the participants the day before the small-group meetings, which offered the possibility of personal acquaintance: this increased trust and acceptability.

The third strength is that the qualitative data was validated by its trustworthiness (17). As to credibility, we used qualitative content analysis as suitable for our aim and there was ongoing collaboration among the three authors. We employed triple coding and met regularly during the comprehensive discussion of categorization. Validity was increased by each author independently reading the notes and discussing them until agreement was attained. Regarding dependability, as the project lasted only six months, our data are presumed to reduce the risk of change in the analysis. To ensure transferability, we informed potential participants orally and in writing about the intervention, and the local coaches got back the consensus and referred this to us. In the present pilot study, we have studied the intervention performed in five Swedish municipalities in Västernorrland, findings may not be applicable in other parts of Sweden as well as in other countries with different reception programs.

Limitations were the large geographical distance between the authors and the local coaches, interpreters and participants. This was not optimal when large groups from different municipalities participated online. A further limitation was the short time (6 months) and out of our control to plan, perform and evaluate the study. Another limitation of the explorative part of the study was that representativity and social desirability could not be studied. There was no use of standardized measures to assess anxiety and stress which is a limitation but due to the limited project time of sex months. But the measures had been taken before and with satisfactory result. Also, there was a limitation of avoidance among the dropouts. Dolezal et al. (22) studied differences in posttraumatic and psychosocial outcomes among refugees, asylum seekers and internally displaced persons, that asylum seekers' uncertainty may include a greater threat, exacerbating posttraumatic beliefs that may work social disconnection. We could not follow up dropouts to study avoidance behaviour but will pay attention to that in future studies.

### Implications

Primary prevention for newcoming refugees is important, as trauma experience generates chronic disease by direct effects and mental illness (depression, PTSD; anxiety, pain), and indirect effects and impaired lifestyle (9). Regarding future refugee support services, particularly in an online format, is recommended to consider a combination online of small, closed group meetings with the possibility of personal acquaintance, trust and individual follow-up psychosocial support for those in need to be paid attention to.

### Future research

Under to the EU mass refugee directive on the time limitation for stay (4 March 2026), the Tidal Agreement emphasizes (2022) and the build-up of infrastructure in Ukraine. Also, train-the-trainer trauma support with local psychologists is needed to plan, perform, and evaluate this short trauma-focused group intervention with group meetings in small groups. It would be of great value for primary prevention to develop the possibility of personal acquaintance in combination with a mobile app offering individual psychosocial support. To our knowledge, this has yet to be studied.

## Conclusion

Trust and reduced anxiety level changed after the intervention and a combination online of small, closed group meetings with the possibility of personal acquaintance, trust and individual follow-up psychosocial support for those in need needs to be paid attention to for future refugee support services, particular an online format.

## Data Availability

The datasets presented in this article are not readily available because the study included vulnerable participants. Requests to access the datasets should be directed to Solvig Ekblad, solvig.ekblad@ki.se.

## References

[1] Gerlach I Ryndzak O. Ukrainian migration crisis caused by the war. Stud Europejskie-Stud Eur Aff. (2022) 26:17–29. 10.33067/SE.2.2022.2

[2] Kaufman KR Bhui K Katona C. Mental health responses in countries hosting refugees from Ukraine. BJPsych Open. (2022) 8:e87. 10.1192/bjo.2022.55 35361299 PMC9059731

[3] Im H Swan LER. “We learn and teach each other”: interactive training for cross-cultural trauma-informed care in the refugee community. Community Ment Health J. (2022) 58:917–29. 10.1007/s10597-021-00899-2 34618270

[4] Burchill J Pevalin DJ. Demonstrating cultural competence within health-visiting practice: working with refugee and asylum-seeking families. Divers Equal Health Care. (2014) 11(2):151–9. 10.21767/2049-5471.100010

[5] Bryant RA Edwards B Creamer M O’Donnell M Forbes D Felmingham KL The effect of post-traumatic stress disorder on refugees’ parenting and their children’s mental health: a cohort study. Lancet Public Health. (2018) 3:e249–58. 10.1016/S2468-2667(18)30051-3 29731158

[6] Buchcik J Kovach V Adedeji A. Mental health outcomes and quality of life of Ukrainian refugees in Germany. Health Qual Life Outcomes. (2023) 21:23–9. 10.1186/s12955-023-02101-5 36894946 PMC9996949

[7] Rizzi D Ciuffo G Sandoli G Mangiagalli M de Angelis P Scavuzzo G Running away from the war in Ukraine: the impact on mental health and internally displaced persons (IDPs) and refugees in transit in Poland. Int J Environ Res Public Health. (2022) 19:16439. 10.3390/ijerph192416439 36554321 PMC9778520

[8] Galatzer-Levy IR Huang SH Bonanno GA. Trajectories of resilience and dysfunction following potential trauma: a review and statistical evaluation. Clin Psychol Rev. (2018) 63(7):41–55. 10.1016/j.cpr.2018.05.008 29902711

[9] Mollica RF Brooks RT Ekblad S McDonald L. The new H^5^ model of refugee trauma and recovery. In: Lindert J Levav I, editors. Violence and Mental Health. NY: Springer Press, NY (2014). p. 341–378.

[10] Ekblad S Gramatik O Suprun Y. Increasing perceived health and mental health literacy among separated refugee Ukrainian families with urgent needs occasioned by invasion—a group intervention study with participatory methodology in Sweden. Front Public Health. (2024) 12:1356605. 10.3389/fpubh.2024.1356605 38799690 PMC11122463

[11] Sijbrandij M Acarturk C Bird M Bryant RA Burchert S Carswell K Strengthening mental health care systems for Syrian refugees in Europe and the Middle East: integrating scalable psychological interventions in eight countries. Eur J Psychotraumatol. (2017) 8(sup2):1–11. 10.1080/20008198.2017.1388102 PMC5687806 29163867

[12] Abtahi Z Potocky M Eizadyar Z Burke SL Fava NM. Digital interventions for the mental health and well-being of international migrants. A systematic review. Res Social Work Pract. (2023) 33(5):518–29. 10.1177/10497315221118854

[13] von Elm E Altman DG Egger M Pocock SJ Gøtzsche PC Vandenbroucke JP. The strengthening the reporting of observational studies in epidemiology (STROBE) statement: guidelines for reporting observational studies. Lancet. (2007) 370(9596):1453–7. 10.1016/S0140-6736(07)61602-X 18064739

[14] Silove D. The psychosocial effects of torture, mass human rights violations, and refugee trauma: toward an integrated conceptual framework. J Nerv Ment Dis. (1999) 187(4):200–7. 10.1097/00005053-199904000-00002 10221552

[15] Leask CF Sandlund M Skelton DA Altenburg TM Cardon G Chinapaw MJM Framework, principles and recommendations for utilizing participatory methodologies in the co-creation and evaluation of public health interventions. Res Involvment Engagement. (2019) 5(2):4–16. 10.1186/s40900-018-0136-9 PMC6327557 30652027

[16] World Health Organization. Translation protocol, November 2003. Appendix 2. Available online at: https://www.cdc.gov/nchs/data/washington_group/meeting6/appendix2_translation.pdf (Accessed April 02, 2024).

[17] Graneheim UH Lundman B. Qualitative content analysis in nursing research: concepts, procedures, and measures to achieve trustworthiness. Nurse Educ Today. (2004) 24(2):105–12. 10.1016/j.nedt.2003.10.001 14769454

[18] Seagle EE Dam AJ Shah PP Webster JL Barrett DH Ortmann LW Research ethics and refugee health: a review of reported considerations and applications in published refugee health literature, 2015–2018. Confl Health. (2020) 14:39. 10.1186/s13031-020-00283-z 32577125 PMC7305588

[19] Piotrowicz K Semeniv S Kupis R Ryś M Perera I Gryglewska B Disease burden in older Ukrainian refugees of war: a synthetic reanalysis of public records data. Lancet Healthy Longevity. (2022) 3:e667–73. 10.1016/S2666-7568(22)00187-8 36122579

[20] Bergevi J Andermo S Woldamanuel Y Johansson U-B Hagströmer M Rossen J. User perceptions of eHealth and mHealth services promoting physical activity and healthy diets: systematic review. JMIR Hum Factors. (2022) 9(2):e34278. 10.2196/34278 35763339 PMC9277535

[21] Voth M Chisholm S Sollid H Jones C Smith-MacDonald L Brémault-Phillips S. Efficacy, effectiveness and quality of resilience-building mobile health apps for military, veteran and public safety personnel populations: scoping literature review and app evaluation. JMIR Mhealth Uhealth. (2022) 10(1):e26453. 10.2196/26453 35044307 PMC8811698

[22] Dolezal ML Alsubaie MK Sheikh I Rosencrans P Walker RS Zoellner LA Differences in posttraumatic and psychosocial outcomes among refugees, asylum seekers and internally displaced persons. J Nerv Ment Dis. (2021) 209(1):28–34. 10.1097/NMD.0000000000001248 33093357

